# Development of a Genome-Edited Tomato With High Ascorbate Content During Later Stage of Fruit Ripening Through Mutation of *SlAPX4*

**DOI:** 10.3389/fpls.2022.836916

**Published:** 2022-04-12

**Authors:** Ju Hui Do, Seo Young Park, Se Hee Park, Hyun Min Kim, Sang Hoon Ma, Thanh Dat Mai, Jae Sung Shim, Young Hee Joung

**Affiliations:** School of Biological Sciences and Technology, Chonnam National University, Gwangju, South Korea

**Keywords:** tomato, genome editing, *SlAPX*, CRISPR/Cas9, ascorbate

## Abstract

Ascorbate is an essential antioxidant substance for humans. Due to the lack of ascorbate biosynthetic enzyme, a human must intake ascorbate from the food source. Tomato is one of the most widely consumed fruits, thus elevation of ascorbate content in tomato fruits will improve their nutritional value. Here we characterized *Solanum lycopersicum ASCORBATE PEROXIDASE 4* (*SlAPX4*) as a gene specifically induced during fruit ripening. In tomatoes, ascorbate accumulates in the yellow stage of fruits, then decreases during later stages of fruit ripening. To investigate whether *SlAPX* is involved in the decrease of ascorbate, the expression of *SlAPX*s was analyzed during fruit maturation. Among nine *SlAPX*s, *SlAPX4* is the only gene whose expression was induced during fruit ripening. Mutation of *SlAPX4* by the CRISPR/Cas9 system increased ascorbate content in ripened tomato fruits, while ascorbate content in leaves was not significantly changed by mutation of *SlAPX4*. Phenotype analysis revealed that mutation of *SlAPX4* did not induce an adverse effect on the growth of tomato plants. Collectively, we suggest that *SlAPX4* mediates a decrease of ascorbate content during the later stage of fruit ripening, and mutation of *SlAPX4* can be used for the development of genome-edited tomatoes with elevated ascorbate content in fruits.

## Introduction

Ascorbate (L-ascorbic acid, L-threo-hex-2-enono-1,4-lactone) is one of the essential antioxidant molecules in plants and animals (Locato et al., 2013; Fenech et al., 2019). While many animals and plants can synthesize ascorbate, humans have lost the ability to synthesize ascorbate due to the mutations that occurred on the L-gulono-1,4-lactone oxidase, which is the last committed enzyme of the pathway (Drouin et al., 2011). Thus, humans must intake ascorbate through diets such as fresh vegetables and fruits. Tomato has been the most produced fruit in the world during the last 20 years. The total production of tomato fruits has been 182 million tons in 2018 and the annual production of tomatoes has increased during the last years (FAOSTAT., 2019). Even though the tomato is the most widely consumed fruit in the world, the tomato has been considered a moderate source of ascorbate due to its relatively low ascorbate content (Gest et al., 2013; Mellidou et al., 2021). Therefore, increasing ascorbate concentration in tomato fruits will have a great impact on human nutrition.

In plants, ascorbate can be synthesized through four different pathways, namely, the D-mannose/L-galactose pathway, the D-glucose pathway, the myo-inositol pathway, and the D-galacturonate pathway (Wheeler et al., 1998; Ishikawa et al., 2018; Paciolla et al., 2019). Among these four pathways, the D-mannose/L-galactose pathway has been recognized as a major ascorbate biosynthetic pathway in plants (Fenech et al., 2019). D-mannose/L-galactose pathway uses D-fructose-6-phosphate as a precursor for ascorbate production. Specifically, D-fructose-6-phosphate is transformed into D-mannose-1-phosphate by phosphomannose isomerase (PMI) and phosphmannomutase (PMN) (Qian et al., 2007; Maruta et al., 2008). GDP-D-mannose pyrophosphorylase (GMP) transfers guanosine monophosphate into D-mannose-1-phosphate to form GDP-D-mannose (Conklin et al., 1999). Then, GDP-D-mannose-3′,5′-epimerase (GME) converts GDP-D-mannose into GDP-L-galactose (Gilbert et al., 2009). GDP-L-galactose is converted into L-galactono-1,4-lactone by GDP-L-galactose-phosphorylase (GGP), L-galactose-1-phosphate phosphatase (GPP), and L-galactose dehydrogenase (L-GalDH) (Gatzek et al., 2002; Laing et al., 2004; Dowdle et al., 2007). The L-galactono-1,4-lactone is finally converted into ascorbate by L-galactono-1,4-lactone dehydrogenase (GLDH) (Wheeler et al., 2015). On the other hand, there exists an ascorbate degradation pathway in plants (Foyer and Noctor, 2011; Locato et al., 2013). Ascorbate is oxidized into monodehydroascorbate (MDHA) by ascorbate peroxidase (APX) and spontaneously converted into dehydroascorbate (DHA). DHA has then reduced again into ascorbate *via* dehydroascorbate reductase (DHAR) or degraded *via* DHA oxidation (Parsons et al., 2011; Caverzan et al., 2012), indicating that oxidation of ascorbate to DHA, which is committed by APX, is required for its degradation in tomato.

Increasing ascorbate content in plants can be achieved by either activation of its biosynthesis or inactivation of its degradation. Most of the attempts have been focused on ectopic expression of ascorbate biosynthetic genes in plants (Agius et al., 2003; Bulley et al., 2012; Macknight et al., 2017; Li et al., 2019). For example, heterogeneous expression of *GGP* significantly increases ascorbate content in potatoes, tomatoes, and strawberries (Bulley et al., 2012). Similarly, overexpression of other ascorbate biosynthetic genes such as *GMP, GME*, and *GPP* leads to a higher accumulation of ascorbate in rice plants (Zhang et al., 2015). Recently, it is also reported that pyramiding of transgenic tomato plants simultaneously overexpressing four ascorbate biosynthetic genes (*GME, GMP, GGP*, and *GPP*) increased ascorbate content up to 18% in tomato fruits (Li et al., 2019). In addition, activation of ascorbate recycling by overexpression of wheat *DHAR* increased ascorbate content in tobacco and maize plants (Chen et al., 2003). Even though activation of ascorbate biosynthesis or recycling greatly increases ascorbate content in plants, it often caused developmental defects due to metabolic perturbations (Bulley et al., 2012; Fenech et al., 2019). To minimize unintended effects by ectopic expression of ascorbate metabolic genes on plants development, suppression of ascorbate degradation genes, such as *APX*s, has been attempted in several plant species (Zhang et al., 2011; Chatzopoulou et al., 2020).

Ascorbate peroxidase (APX) plays an important role in protecting plants from oxidative stress conditions produced during normal developmental processes and stress responses through the enzymatic conversion of H_2_O_2_ into H_2_O using ascorbate as an electron donor (Koussevitzky et al., 2008; Guo et al., 2020). Due to its importance for plant development and adaptation to the environment, the downregulation of *APX* also occasionally caused a negative effect on plants growth (Pnueli et al., 2003; Locato et al., 2013). For example, knockout mutation of Arabidopsis *AtAPX1* showed retarded growth and reduced stress tolerance with elevated hydrogen peroxide level (Pnueli et al., 2003). Plants possess multiple APX isozymes. They have localized either mitochondria, chloroplasts, peroxisomes, or cytosol (Shigeoka et al., 2002; Najami et al., 2008; Yin et al., 2019; Tyagi et al., 2020; Li et al., 2021), and *SlAPX* isozymes showed different kinetic properties such as optimal pH, Km, and Vmax (Marquez et al., 1996; Liu et al., 2019; Wu and Wang, 2019). In addition, their expression levels were differentially regulated in plants. These results imply that each *APX* might play distinguished functions during plant growth and stress responses. To improve ascorbate content in tomato plants with minimal alteration on plant growth, identification of the *SlAPX* specifically acting during fruit maturation is crucial. In tomatoes, there exist nine putative *SlAPX*s containing three cytosolic, two peroxisomal, two chloroplastic, and two uncharacterized *APX*s (Najami et al., 2008). Here, we aimed to develop tomato plants with elevated ascorbate content in fruits through mutation of the fruit-specific *SlAPX* by CRISPR/Cas9-mediated mutagenesis. Among nine *SlAPX*s, *SlAPX4* is the only gene showing fruit-specific expression patterns. Thus, we selected *SlAPX4* as a target for CRISPR/Cas9-mediated mutagenesis and analyzed the effect of *SlAPX4* mutation on ascorbate content, expression of ascorbate metabolic genes, and development of tomato plants.

## Materials and Methods

### Plant Growth Conditions

Tomato seeds (*Solanum lycopersicum* cv. Micro-Tom) used in this study were kindly provided by prof. Sanghyeob Lee in the Sejong University in Korea. Tomato seeds were sown on a Murashige–Skoog (MS) solid medium [4.4 g/l of MS salt (Duchefa, Netherlands), 30 g/l of sucrose (Sigma-Aldrich, USA), and 0.5 g/l of 2-(N-morpholino) ethanesulfonic acid (MES) (Duchefa, Netherlands), pH 5.7] and incubated in a growth room with the light-dark cycle of 16 h light/8 h dark at 22 to 24°C for gene expression analysis and agrobacterium-mediated transformation. For growing tomato plants until fruit ripening stage, tomato seeds (*S. lycopersicum* cv. Micro-Tom) were sown on a soil pot supplemented with a complex fertilizer Green coat (DHC, Japan) and incubated in a growth room with the light-dark cycle of 16 h light/8 h dark at 22 to 24°C with 65% of relative humidity until maturity. Plants were fully watered at 2-day intervals. Each pot contained one tomato plant and the pots were randomly distributed in the growth room. An 8-week-old tomato plants were used for gene expression analysis and ascorbate quantification in leaves. A 10-week-old tomato plants were used for phenotypic analysis. For analyzing the expression level of ascorbate metabolic genes and ascorbate content in fruits, three fruits per plant from three independent plants were collected at the stage of mature green, breaking, yellowing, and red ripening.

### Plasmid Construction

To target the *SlAPX4* coding region for CRISPR/Cas9-mediated mutagenesis, four independent single-guide RNAs (sgRNAs) were cloned into the pAGM4723 vector using the Golden gate system as previously described (Engler et al., 2009). The *SlAPX4-*specific sgRNAs were amplified with a forward primer (sgRNA1; 5′-TGTGGTCTCAATTTTTGGAAATCGACGTTTGATGTTTTAGAGCTAGAAATAGCAAG-3′, sgRNA2; 5′-TGTGGTCTCAATTAAGCAGTTGAAAAATGTAAGGTTTTAGAGCTAGAAATAGCAAG-3′, sgRNA3; 5′-TGTGGTCTCAATTGTTAAGAATTTTTTACATGAGTTTTAGAGCTAGAAATAGCAAG-3′, and sgRNA4; 5′-TGTGGTCTCAATTGATGTCAAAACCAAAACTGGGTTTTAGAGCTAGAAATAGCAAG-3′) and a reverse primer (CRISPR Universal, 5′-TGTGGTCTCAAGCGTAATGCCAACTTTGTAC-3′) using the pICH86966 plasmid as a template. The PCR products and *U6* promoter of pICSL01009 plasmids were cloned into the pICH47751 plasmid through BasI (NEB, USA) restriction site. The pICH47751 harboring sgRNA under the control of the *U6* promoter, pICH47732_NPTII, and pICH47742_35S:Cas9 plasmids were digested with BpiI (Thermo Scientific, USA), and integrated into pAGM4723 vector using T4 Ligase (NEB, USA). The resultant pAGM4723 harboring *SlAPX4*-specific sgRNA was used for transient expression analysis and agrobacterium-mediated transformation.

### Transient Expression in Tomato Plants

The four plasmids harboring different sgRNAs were transformed into *Agrobacterium tumefaciens* strain GV3101. The transformed GV3101 were incubated in yeast extract peptone (YEP) liquid media [10 g/l of yeast extract (Sigma-Aldrich, USA), 10 g/l of peptone (Sigma-Aldrich, USA), and 5 g/l of NaCl (Sigma-Aldrich, USA), pH 7.2] supplemented with 50 mg/l rifampicin (Duchefa, Netherlands) and 50 mg/l kanamycin (Biosesang, Korea) for overnight at 28°C. In total, 1 ml of overnight culture was transferred into 20 ml fresh YEP media supplemented with 50 mg/l rifampicin and 50 mg/l kanamycin and incubated for 16 h at 28°C. The bacterial solution was centrifuged at 3,500 rpm for 20 min, and the resultant bacterial pellet was resuspended in 10 mM MgCl_2_ (Sigma-Aldrich, USA) solution supplemented with 200 μM acetosyringone (Sigma-Aldrich, USA) (final OD_600_ = 0.8–0.9) and incubated at room temperature for 3 h. The bacterial solution was infiltrated into tomato cotyledons using a needless syringe. The ~50 infiltrated cotyledons were harvested 5 days after infiltration for genomic DNA extraction and further PCR analysis. The efficiency of four sgRNAs was evaluated by sequencing analysis of TA-cloned PCR products.

### Agrobacterium-Mediated Tomato Transformation

The final construct harboring *SlAPX4*-specific sgRNA and SpCas9 was transformed into *A. tumefaciens* strain LBA4404 for agrobacterium-mediated transformation. The agrobacterium harboring the plasmids were incubated in YEP liquid media containing 50 mg/l rifampicin and 50 mg/l kanamycin overnight at 28°C. A total of 2 ml of the overnight culture solution was transferred into 3 ml of YEP media supplemented with 50 mg/l rifampicin and 50 mg/l kanamycin and incubated at 28°C until final OD_600_ was between 0.7 and 0.8. The solution was centrifuged at 3,100 rpm for 20 min and the resultant pellet was resuspended in half strength of MS liquid media (2.2 g/l of MS salt, 20 g/l of sucrose, and 0.5 g/l of MES, pH 5.7) containing 200 μM acetosyringone for 4 h. The Agrobacterium solution was then cocultivated with tomato cotyledons for 10 min. After cocultivation, tomato cotyledons were placed upside down on coculture medium (4.4 g/l of MS salt, 30 g/l of sucrose, 8 g/l of plant agar, 0.1 mg/l of indole-3-acetic acid (IAA), 2 mg/l of zeatin, and 200 μM acetosyringone, pH 5.2) and incubated in the dark for 2 days. Then the tomato cotyledons were transferred into shoot induction medium (4.4 g/l of MS salt, 30 g/l of sucrose, 8 g/l of plant agar, 0.1 mg/l of IAA, 2 mg/l of zeatin, 50 mg/l of kanamycin, and 250 mg/l of cefotaxime, pH 6.0) and incubated until the shoot has appeared. After complete formation of the shoot, the callus was incubated on root induction medium (4.4 g/l of MS salt, 15 g/l of sucrose, 8 g/l of plant agar, 2 mg/l of indolebutyric acid, 50 mg/l of kanamycin, and 250 mg/l of cefotaxime, pH 6.0). The regenerated seedlings with shoots and roots were then transferred into the soil for further growth.

### Analysis of Mutation Pattern in Transgenic Plants

To analyze the mutation patterns, the *SlAPX4* genomic region targeted by a sgRNA was amplified by PCR using the gene-specific primers (Supplementary Table S1) with genomic DNAs extracted from leaves of non-transgenic and transgenic plants. Plant genomic DNA preparation was carried out as follows. A total of 500 mg of tomato leaves were homogenized by a mortar and a pestle with liquid N_2_. The ground sample was resuspended with plant genomic DNA extraction buffer [50 mM Tris-Cl (pH 7.6), 17 mM sodium dodecyl sulfate, 100 mM NaCl, 50 mM ethylenediaminetetraacetic acid (pH 7.6), 1% β-mercaptoethanol, and 42 mg/l RNase A] and incubated at 55°C for 10 min. A total of 500 μl of phenol: chloroform: isoamyl alcohol (25:24:1) was added into the extract and centrifuged at 13,000 rpm at 4°C for 15 min. The supernatant was transferred into a new microcentrifuge tube and one volume of isopropanol was added to the solution. The mixture was centrifuged at 13,000 rpm at 4°C for 10 min. The resultant pellet obtained after centrifugation was dissolved in 50 μl of sterilized distilled water and used as a template for a PCR reaction. PCR conditions were as follows: 95°C for 10 min and 40 cycles of 95°C for 30 s, 54°C for 30 s, and 72°C for 1 min. PCR products were then purified using the QIAquick PCR Purification Kit (Qiagen, Germany) and used for further direct sequencing, targeted deep sequencing, and cloning into the pGEM-T Easy vector (Promega, USA).

### Ribonucleic Acid Isolation and Quantitative Real-Time PCR Analysis

Total RNA was extracted from leaves using an RNeasy Plant Mini Kit (Qiagen, Germany) according to the manufacturer's instruction. A total of 1 μg of total RNA was treated with an RNase-free DNase I (Qiagen, Germany), and used for first-strand synthesis reactions using the QuantiTect Reverse Transcription Kit (Qiagen, Germany) according to the manufacturer's instruction. The products were used for quantification of target transcripts through quantitative real-time PCR (qRT-PCR) analysis with gene-specific primers. The tomato *SlACTIN* (*SlACT*, Solyc03g078400) was used as an internal control for normalization. Gene expression and ascorbate content were analyzed from three biological replicates. The primer information used for qRT-PCR gene expression is given in Supplementary Table S1.

### Ascorbate Quantification

Endogenous ascorbate content was quantified using the Ascorbic Acid Assay Kit (Abcam, UK) in fruits and high-performance liquid chromatography (HPLC) analysis in leaves. For ascorbate quantification in fruits, 40 mg of samples were rinsed with ice-cold phosphate-buffered saline (PBS) pH 7.4. The rinsed samples were homogenized with 400 μl of ddH_2_O using TissueLyzer II (Qiagen, Germany). The crude extract was centrifuged at 13,000 rpm at 4°C for 5 min, and the supernatant was used for ascorbate quantification assay. Further analysis and quantification of ascorbate were performed according to manufacturer's instruction. Endogenous ascorbate content in leaves was determined by an HPLC analysis. A total of 0.01 g of plant powder ground with liquid nitrogen was homogenized in 3 ml of 5% meto-phosphate acid (MPA) (Sigma-Aldrich, USA). The homogenate was centrifuged at 13,000 rpm at 4°C for 10 min. The supernatant was filtered using a syringe filter (0.45 um, Biofact, Korea). The flow-through was incubated with 2 mg of dithiothreitol (DTT) in the dark for 25 min. Separation was achieved by isocratic elution with 0.1% trifluoroacetic acid (TFA) (pH 1.88). A total of 10 μl of the reaction mixture was injected into a Gemini C18 column (150 mm ×4.6 mm, particle size 5 μm; Phenomenex, USA) and flowed at a rate of 1 ml/min for 10 min. The column temperature was maintained at 30°C and the autosampler temperature was maintained at 8°C. Ascorbate was detected with photodiode array (PDA) at 244 nm. Quantification of ascorbate was performed using LabSolutions CS software (Shimadzu, Japan). The amount of ascorbate was determined from three biological replicates using the standard curve and normalized to the mass of fresh plant tissue.

### Phenotype and Agronomic Trait Analysis

Ten mutant plants per line were used for phenotypic analysis of *slapx4* mutants. The height was measured from the bottom of the pot to the top of the plant. The number of buds, fruits, and seeds per was counted from each individual plant (*n* = 10).

### Statistics

The statistical significance of gene expression and metabolite content between Micro-Tom wild-type (WT) plants and *slapx4* mutant plants with one-way ANOVA test using GraphPad Prism version 8.0.1 (https://www.graphpad.com/scientific-software/prism/). Three biological replicates were used for gene expression and ascorbate quantification. Ten independent tomato plants were used for phenotypic analysis (plant height, flower number, fruit number, and seed number).

## Results

### Identification of the Fruit-Specific *SlAPX* in Tomato

**Figure 1:**
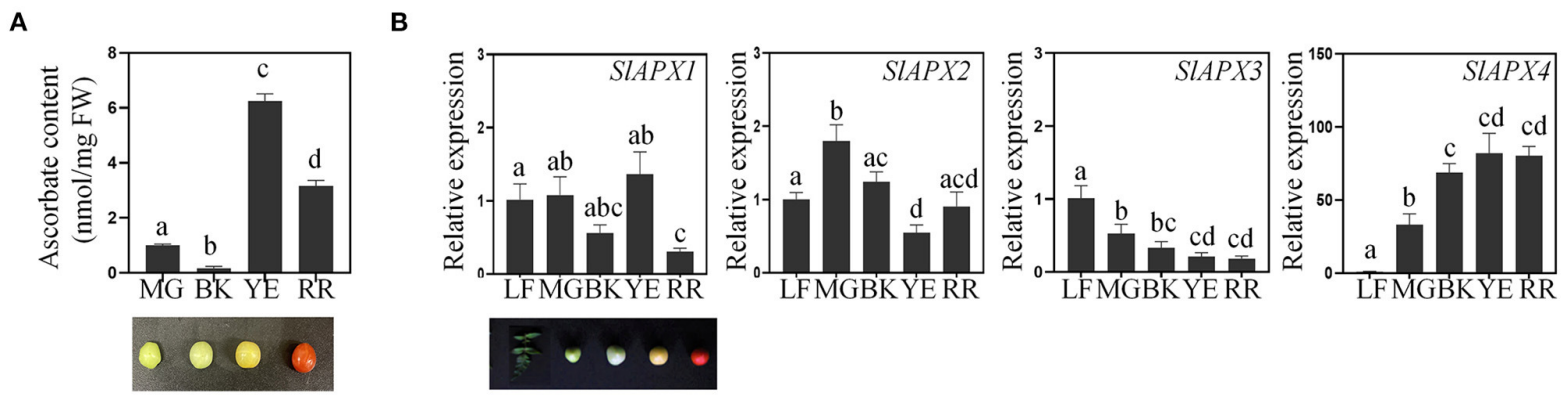
Regulation of ascorbate accumulation during fruit development. **(A)** Change of ascorbate content during tomato fruit ripening. Ascorbate content (nmol per gram fresh weight) was determined from fruits at the indicated developmental stages. **(B)** The relative expression level of *SlAPX*s during fruit ripening. *SlACT* (Soly03g078400) was used as an internal control for normalization. Three tomato fruits from three individual plants at the indicated developmental stages were pooled for ascorbate quantification and gene expression analysis. Data represent the mean value + SD of three biological replicates. A significant difference was indicated with different letters above the bars (one-way ANOVA test). FW, fresh weight; MG, mature green; BK, breaking; YE, yellowing; RR, red ripening; LF, leaves.

To determine temporal regulation of ascorbate accumulation in tomato plants, we monitored changes in ascorbate content during fruit maturation. Ascorbate content was lower in the mature green (MG) and breaking (BK) stage than yellowing (YE) and red-ripening (RR) stages of tomato fruits (Figure 1A). The ascorbate content peaked in the YE stage then decreased in the RR stage. These results indicated that ascorbate is accumulated during the early stage of fruit maturation then decreased during the later stage of fruit maturation. To explain the observed changes through transcriptional regulation of the ascorbate metabolic pathway, we first analyzed the expression pattern of genes that are involved in ascorbate biosynthesis in tomatoes (Supplementary Figure S1). Gene expression analysis revealed that ascorbate biosynthetic genes (*SlGMP, SlGLDH*, and *SlGME2*), except *SlGME1*, showed the highest expression level in leaves. During fruit maturation, *SlGMP* and *SlGLDH* were highly expressed in mature green and breaking stages of fruit development, then the expression level was decreased in yellowing and red ripening stages of fruit development (Supplementary Figure S1). Expression of *SlGME1* was lower in the red ripening stage than other stages of fruit. The expression level of ascorbate recycling genes (*SlDHAR1, SlDHAR2*, and *SlMDHAR*) was higher in the early stage of fruit maturation than the later stage of fruit maturation (Supplementary Figure S1). We then checked the expression pattern of *SlAPX* genes during fruit maturation (Figure 1B and Supplementary Figure S2). Among the nine *SlAPX* genes, four *SlAPX*s (*SlAPX1, 2, 3*, and *4*) were mainly expressed in tomato plants (Sato et al., 2012) (Supplementary Figure S2). *SlAPX1, 2*, and *3* were expressed in several tomato tissues, namely, leaves, roots, flowers, and fruits, while *SlAPX4* was predominantly expressed in fruits (Figure 1B and Supplementary Figure S2). Expression of *SlAPX1, 2*, and *3* was generally higher during the early stage of fruit ripening (Figure 1B) while *SlAPX4* expression was gradually upregulated during fruit ripening (Figure 1B). Based on these data, we chose *SlAPX4* as a target of mutagenesis for fruit-specific accumulation of ascorbate in tomatoes.

### Generation of CRISPR/Cas9-Mediated *slapx4* Mutants

**Figure 2:**
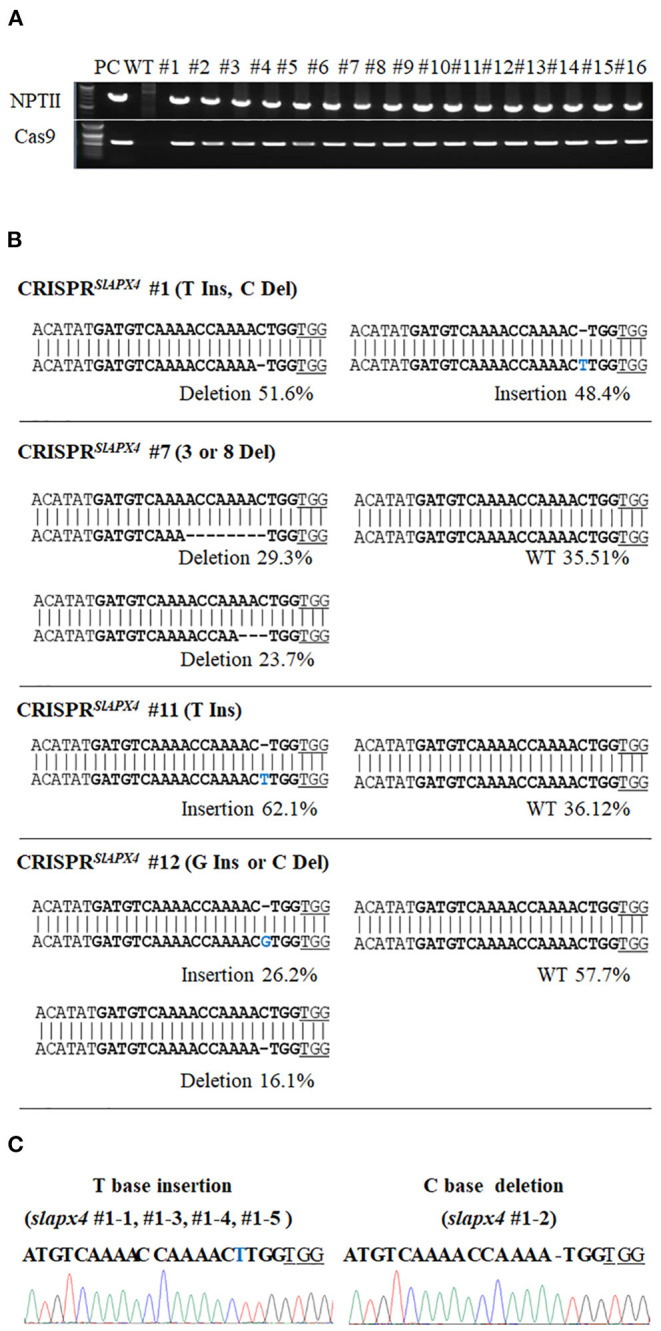
Generation of *SLAPX4* mutant plants. **(A)** Validation of transgenic plants using *NPTII* and *Cas9*-specific PCR reactions. **(B)** Sequencing analysis of *SlAPX4* locus targeted by a single-guide RNA (sgRNA). Genomic DNA extracted from the T_0_ stage of CRISPR^*SlAPX*4^ transgenic tomato plants was used for amplification of the *SlAPX4* genomic region targeted by a sgRNA through PCR reactions. The PCR products were then applied for targeted deep sequencing analysis. **(C)** A representative chromatograph of edited alleles in the T_1_ stage of *SLAPX4* mutant plants was shown with the mutated target region. Bold, sgRNA target site; blue, base insertion; underlined, PAM site.

Since *SlAPX4* is the only gene, whose expression is upregulated during the later stage of fruit ripening, mutation of *SlAPX4* might slow down the decrease of ascorbate content during the later stage of fruit ripening. To address the possibility, we generated *slapx4* mutant plants by applying clustered regularly interspaced short palindromic repeats (CRIPSR)/CRISPR-associated protein 9 (Cas9) system in tomatoes. To screen a sgRNA that effectively induces mutation on the *SlAPX4* gene, we generated four plant expression vectors that encode independent single guide RNAs (sgRNAs) targeting the exon region of *SlAPX4*. The expression vectors were infiltrated into tomato cotyledons to determine the efficiency of each sgRNAs. The mutation efficiency of each sgRNA was determined by sequencing the genomic region targeted by a sgRNA. Among the tested four independent sgRNAs, the sgRNA4 that targets the first exon of *SlAPX4* showed the highest mutation efficiency (Table 1). Thus, the plant expression vector harboring SpCas9 and sgRNA4 was introduced into tomato plants to generate *slapx4* mutant plants. A total of 16 T_0_ plants (CRISPR/Cas9^*SlAPX*4^) were selected based on antibiotic resistance and PCR verification of *NPTII* and *Cas9* genes (Figure 2A). Among the 16 T_0_ plants, four plants contained mutation on the *SlAPX4* genomic region targeted by sgRNA4. Specifically, one heteroallelic mutant (CRISPR/Cas9^*SlAPX*4^ #1, T insertion/ C deletion), one monoallelic mutant (CRISPR/Cas9^*SlAPX*4^ #11, T insertion), and two mosaic mutants (CRISPR/Cas9^*SlAPX*4^ #7 and #12) were identified by sequencing analysis (Figure 2B and Supplementary Figure S3). Amino acid analysis revealed that either T insertion or C deletion caused a loss of multiple catalytic residues and cation-ligand residues with early termination (Table 2). Thus, CRISPR/Cas9^*SlAPX*4^ #1 and CRISPR/Cas9^*SlAPX*4^ #11 T_0_ plants were further propagated into the T_1_ stage, and three biallelic homozygous *SLAPX4* mutant plants with T base insertion (*SLAPX4* #1-T_ins and #11-T_ins) or C base deletion (*SLAPX4* #1-C_del) were selected for further analysis (Figure 2C).

**Table 1:** Mutation efficiency of sgRNAs targeting *SlAPX4*.

**sgRNA**	**Sequence**	**Total sequenced colonies (number)**	**Colonies with mutation on 3 bp upstream from PAM site (number)**	Mutation efficiency (%)*
sgRNA1	**TTTGGAAATCGACGTTTGAT** CGG	442	0	0
sgRNA2	**AAGCAGTTGAAAAATGTAAG** AGG	273	0	0
sgRNA3	**GTTAAGAATTTTTTACATGA** TGG	548	2	0.36
sgRNA4	**GATGTCAAAACCAAAACTGG** TGG	561	5	0.89

Mutation efficiency of sgRNAs determined by sequencing plasmids containing PCR fragments of genomic regions targeted by single-guide RNA (sgRNA). *Mutation efficiency= number of colonies with mutation on 3 bp upstream from PAM site/number of total sequenced colonies. Bold, sgRNA target site; underlined, PAM site.

**Table 2:** Amino acid sequence analysis of the target site in *slapx4* mutant plants.

**Line**	**Genotype**	**Amino acid sequence**
WT	WT	MVKCYPTVSEEYQKAVEKCKRKLRGLIAEKNCAPIMLRLAWHSAGTYDVKTKTGGPFGTIRHPNELKHGANNGLDIAVRLLEPIKEQFPILSYADFYQLAGVVAVEVTGGPDIPFHPGRQDKTEPPPEGRLPDATKGSDHLREVFGHMGLSDKDIVALSGGHTLGRCHKERSGFEGAWTNNPLIFDNSYFKELLSGEKEGLLQLPSDKALLEDPVFRPLVEKYAADEDAFFADYAEAHLKLSELGFADAE
*slapx4* #1 T ins	T insertion	MVKCYPTVSEEYQKAVEKCKRKLRGLIAEKNCAPIMLRLAWHSAGTYDVKTKTWWSIWNNQAPE*
*slapx4* #1 C del	C deletion	MVKCYPTVSEEYQKAVEKCKRKLRGLIAEKNCAPIMLRLAWHSAGTYDVKTK**MVVHLEQSGTRMNLNMELTM** **VLILLFDFWSRSRNSSQ** SYPTLTFISWLE*
*slapx4* #11 T ins	T insertion	MVKCYPTVSEEYQKAVEKCKRKLRGLIAEKNCAPIMLRLAWHSAGTYDVKTKTWWSIWNNQAPE*

*WT, wild-type; asterisk, stop codon; Bold with underlined, mutated regions; Red, catalytic residues (Najami et al., 2008); Blue, cation-ligand residues (Najami et al., 2008); WT, Micro-Tom wild-type plants*.

### Accumulation of Ascorbate in *slapx4* Mutant Plants

**Figure 3:**
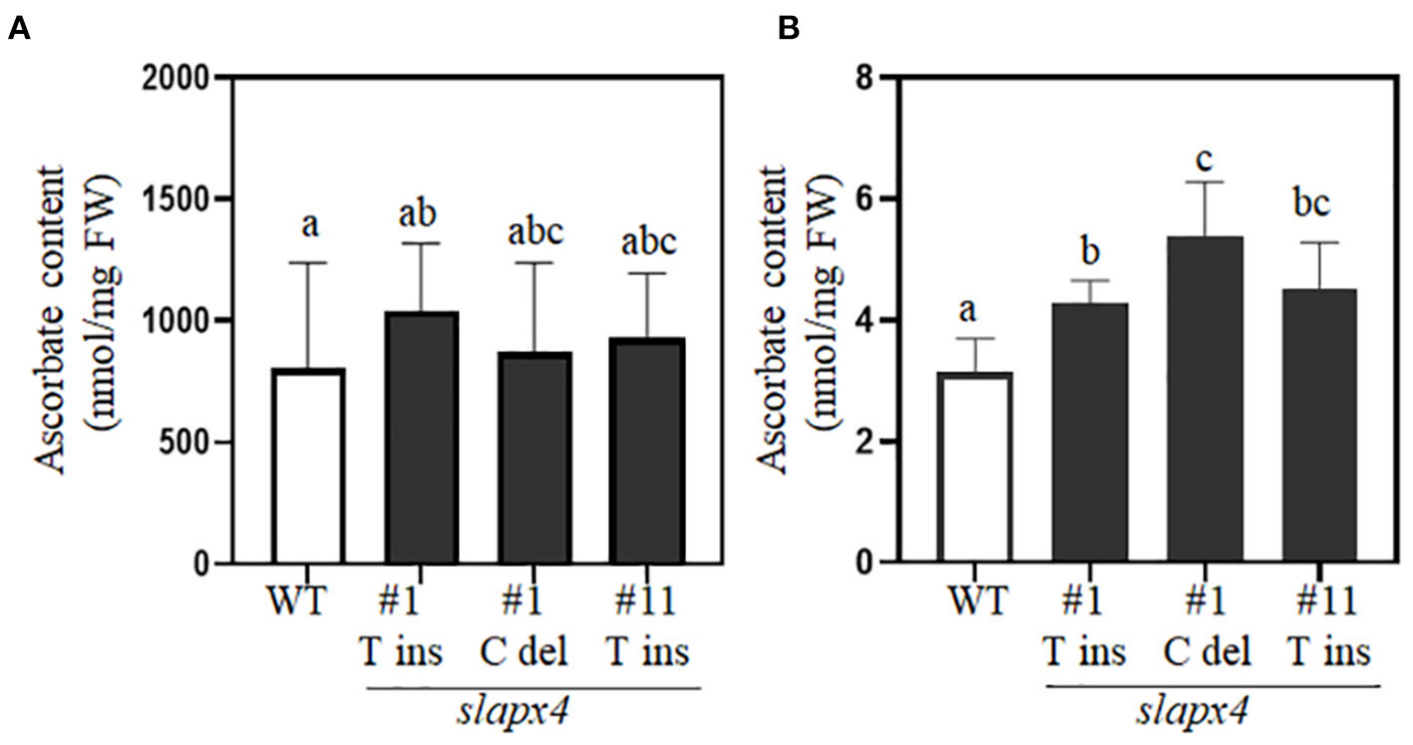
Determination of ascorbate content in *SLAPX4* mutant plants. **(A,B)** Wild-type (WT) and *SLAPX4* mutant plants containing biallelic mutation (T base insertion; *SLAPX4* #1 T ins and #11 T ins, C base deletion; *SLAPX4* #1 C del) were grown in the soil. The plants were used for quantification of ascorbate content (nmol per fresh weight) in leaves **(A)** and red-ripening fruits **(B)**. Leaves and fruits from three independent plants were pooled for ascorbate quantification. Data represent the mean value + SD of three biological replicates. A significant difference was shown with different letters above the bars (one-way ANOVA test). FW, fresh weight.

**Figure 4:**
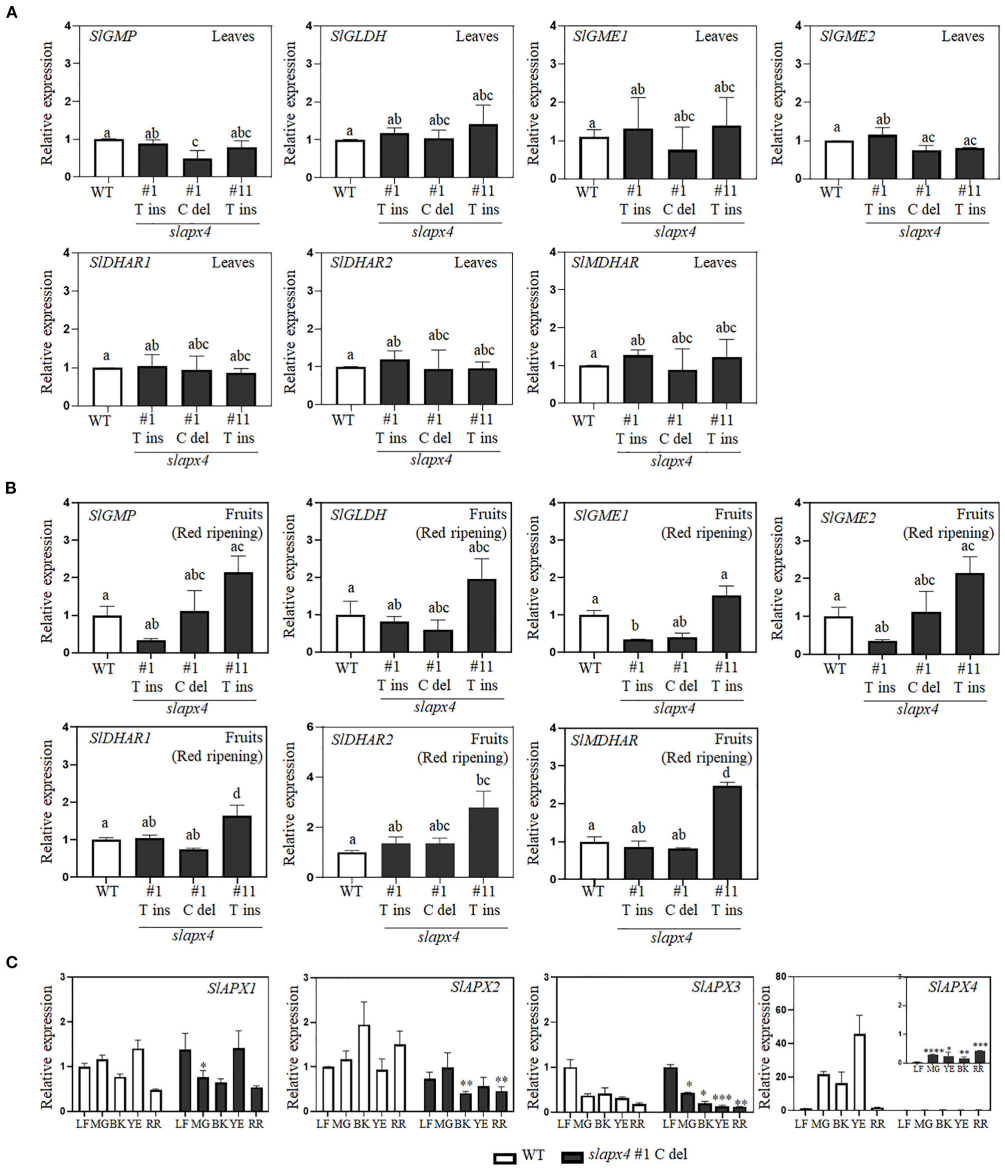
Expression pattern of ascorbate metabolic genes in *SLAPX4* mutant plants. **(A,B)** Relative expression levels of ascorbate metabolic genes in leaves **(A)** and fruits **(B)** of wild-type (WT) and *SLAPX4* mutant plants (*SLAPX4* #1 T ins, #1 C del, and #11 T ins). **(C)** The relative expression of *SlAPX*s in WT and *SLAPX4* mutant plants during fruit maturation. Leaves or fruits from three individual tomato plants at the indicated stages of development were pooled for gene expression analysis. *SlACT* (Soly03g078400) was used as an internal control for normalization. Data represent the mean value + SD of three biological replicates. A significant difference was shown with different letters above the bars for **(A)** (one-way ANOVA test). Significant difference between WT and *SLAPX4* mutant plants was shown by asterisks for **(B)** (Student's *t*-test, *p < 0.05, **p < 0.001, and ***p < 0.0001). LF, leaves; MG, mature green; BK, breaking; YE, yellowing; RR, red ripening.

To investigate the effects of *SlAPX4* mutation on ascorbate accumulation in tomato plants, we analyzed ascorbate content in leaves and fruits of *slapx4* mutant plants (Figure 3). No significant difference was detected on ascorbate content in leaves between WT and *slapx4* mutant plants (Figure 3A). On the other hand, compared with WT control plants, all the selected *slapx4* mutant plants possessing either insertion or deletion mutation on *SlAPX4* accumulated significantly higher levels of ascorbate in fruits (Figure 3B). To investigate the possibility that the change of ascorbate content in *slapx4* mutant plants alters ascorbate metabolic gene expression, we analyzed the expression pattern of four ascorbate biosynthetic genes (*SlGMP, SlGLDH, SlGME*, and *SlGME2*) and three ascorbate recycling genes (*SlDHAR1, SlDHAR2*, and *SlMDHAR*) in leaves and fruits of *slapx4* mutant plants. The expression level of ascorbate biosynthesis and recycling genes in both leaves and fruits was similar between WT and *slapx4* mutant plants (Figures 4A,B). We then checked the expression pattern of *SlAPX*s in WT and *slapx4* mutant plants. Similar to ascorbate biosynthetic genes, the expression of *SlAPX1, 2*, and *3* was similar between WT and *slapx4* mutant plants (Figure 4C and Supplementary Figure S4). On the other hand, compared with WT plants, *SlAPX4* expression was significantly reduced in *slapx4* mutant plants (Figure 4C and Supplementary Figure S4).

### Phenotype Analysis of *slapx4* Mutant Plants

**Figure 5:**
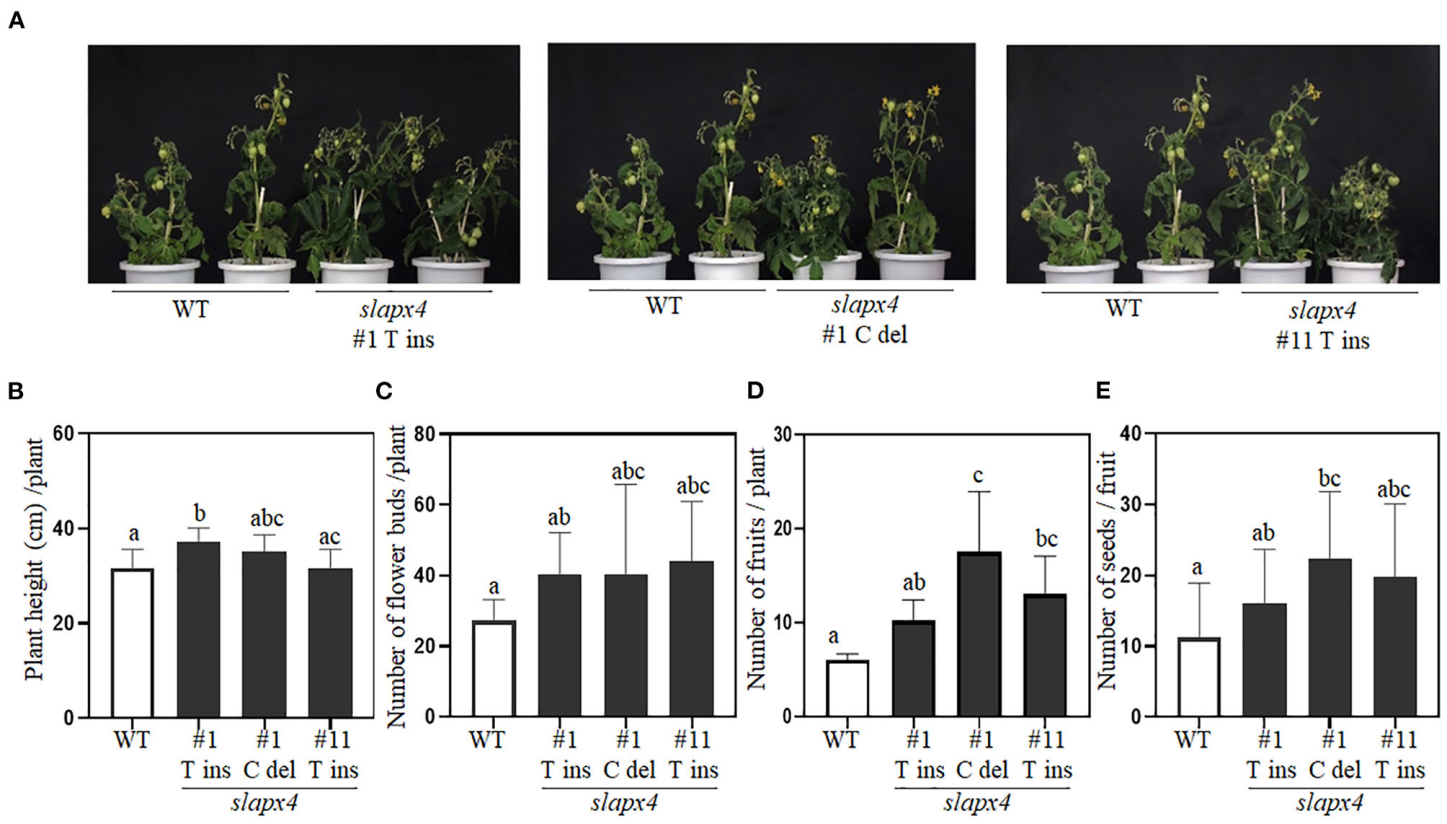
Phenotypic analysis of *SLAPX4* mutant plants. **(A–E)** Wild-type (WT) and *SLAPX4* mutant plants (*SLAPX4* #1 T ins, #1 C del, and #11 T ins) were grown on the soil until maturity. **(A)** The phenotype of the representative plants was visualized by taking a picture at the mature green stage. Same WT plants were used for three independent pictures. Plant height **(B)**, flower bud number **(C)**, fruit number **(D)**, and seed number **(E)** per plant were measured from ten independent plants. Data represent the mean value + SD (*n* = 10). A significant difference was shown with different letters above the bars (one-way ANOVA test).

To investigate the potential effects of *SlAPX4* mutation on plant growth, we monitored the developmental phenotypes of *slapx4* mutants whose red ripening fruit showed higher ascorbate content (Figure 5). Overall, *slapx4* mutant plants did not show growth defects compared with WT control plants (Figure 5A). Total plant height determined at the breaking stage was similar between WT and two of three *slapx4* mutant plants (*slapx4* #1 C_del and #11 T_Ins) (Figure 5B). The number of flower buds was not significantly different between WT and *slapx4* mutant plants (Figure 5C). In addition, the fruit of WT and *slapx4* mutant plants showed similar morphology (Supplementary Figure S5). On the other hand, two of three *slapx4* mutant plants (*slapx4* #1 C_del and #11 T_Ins) produced more fruits (Figure 5D), and *slapx4* #1 C-del plants had a higher number of seeds than WT control plants (Figure 5E).

## Discussion

Tomatoes have relatively low ascorbate content compared with other fruits, such as strawberry and grape (Beecher, 1998). Based on its importance as a human diet and its high raw intake, an increase of ascorbate content in tomato fruits will have far-reaching effects from a nutritional point of view. Here we have suggested the strategy to develop tomatoes with elevated ascorbate content through targeted mutagenesis of fruit-specific *SlAPX4* using the CRISPR/Cas9 system.

Ascorbate content analysis in tomato plants revealed that ascorbate levels increase during the early stages of fruit ripening and decrease during the later stage of fruit ripening (Figure 1A). Similar accumulation patterns were observed in various tomato cultivars originated from different continents (Abushita et al., 1997; Mellidou et al., 2012; Sacco et al., 2019), suggesting that developmental regulation of ascorbate content is well-conserved in many tomato cultivars. Ascorbate pool in fruits is known to be closely correlated with the oxidative status of tomato fruits (Imai et al., 2009; Li et al., 2010; Lima-Silva et al., 2012). The increase of ascorbate content would be beneficial to maintain redox balance during fruit development and ripening through the effective removal of reactive oxygen species (Jimenez et al., 2002; Gapper et al., 2013). However, little is known how ascorbate content increases during tomato fruit ripening. Gene expression analysis showed that expression of ascorbate biosynthetic genes was generally downregulated during fruit maturation (Supplementary Figure S1). These results suggest that accumulation of ascorbate during the early stage of fruit maturation could be accomplished by other mechanisms rather than transcriptional regulation of biosynthetic pathway (Imai et al., 2009; Li et al., 2010). One possible explanation for the phenomenon is the source to sink allocation of ascorbate in plants. It is predicted that translocation of ascorbate from leaves to developing fruits, rather than *de-novo* synthesis, provides an ascorbate pool in fruits at the early stage of fruit ripening (Badejo et al., 2012). It has also been suggested that enhanced rates of ascorbate recycling increased ascorbate content during fruit ripening (Mellidou et al., 2012). Another potential mechanism related to ascorbate accumulation during fruit ripening is a transcriptional regulation of *SlAPX* genes. We found that four *SlAPX*s (*SlAPX1, 2, 3*, and *4*) among nine *SlAPX*s were mainly expressed in tomato plants (Figure 1B and Supplementary Figure S2). Expression of *SlAPX1, 2*, and *3* was gradually reduced during fruit ripening, while *SlAPX4* expression was induced during fruit ripening in tomato plants (Figure 1B). These temporal expressions of *SlAPXs* might be contributed to shaping ascorbate accumulation during fruit ripening.

Mutation of ascorbate peroxidase is a promising strategy for increasing ascorbate content in plants. It has been reported that the tomato cultivar with elaborated ascorbate content showed reduced expression of ascorbate peroxidase (Di Matteo et al., 2010). However, due to its importance for detoxification of hydrogen peroxide with ascorbate (Caverzan et al., 2012), precise functional characterization of ascorbate peroxidase is necessary to minimize potential negative effects caused by the mutation of *APX* on plant development. Expression characteristics of *SlAPX4* suggested it as an attractive target for developing genome-edited tomatoes with elevated ascorbate content in fruits. Unlike other *SlAPX* genes, *SlAPX4* was predominantly expressed in fruits than other tissues such as leaves, roots, and flowers (Figure 1B and Supplementary Figure S2). In addition, *SlAPX4* is the only gene whose expression was strongly expressed during the later stage of fruit ripening. On the other hand, other *SlAPX*s (*SlAPX1, SlAPX2*, and *SlAPX3*) were predominantly expressed during the early stage of fruit development (Figure 1B and Supplementary Figure S2). These results suggest that *SlAPX4* could be related to the decrease of ascorbate content during the later stage of fruit ripening. Ascorbate content analysis revealed that the mutation of *SlAPX4* significantly increased ascorbate content up to 2-fold in red ripening fruits (Figure 3B). Similar to our result, it has been reported that suppression of mitochondrial *APX* expression increases ascorbate content in plants due to lower degradation of ascorbate (Zhang et al., 2011; Chatzopoulou et al., 2020). However, suppression of *APX* expression often caused abnormal growth patterns (Pnueli et al., 2003; Chatzopoulou et al., 2020). These growth alterations could be caused by disruption of the ascorbate metabolic pathway during plant development. Gene expression analysis revealed that the expression level of ascorbate metabolic genes in *slapx4* mutant plants was similar to that of WT control plants in leaves (Figure 4A). In addition, the leaf ascorbate content was not significantly affected by the *SlAPX4* mutation (Figure 3A). Moreover, phenotype analysis of *slapx4* mutant plants revealed that all the generated *slapx4* mutant plants grew normally without growth retardation (Figure 5). These results suggest that *SlAPX4* is mainly involved in ascorbate metabolism during fruit maturation. Interestingly, *slapx4* mutant plants produced slightly more flowers and fruits compared with WT control plants (Figures 5C,D). A similar result was obtained from tomato plants with elevated ascorbate by suppression of ascorbate oxidase (Abdelgawad et al., 2019). These observed growth changes support the idea that the increase of ascorbate content affects plant development through alteration of hormonal signal transduction (Lima-Silva et al., 2012; Akram et al., 2017).

Even though the increase of ascorbate content in fruits is beneficial for human nutrition, the excessive increase of ascorbate content causes developmental defects in fruit shape and seed production (Bulley et al., 2012). Overexpression of the ascorbate biosynthetic gene increases metabolic flux to ascorbate biosynthesis, resulting in the decrease of metabolites required for cell wall biosynthesis (Dumville and Fry, 2003; Fenech et al., 2019). Our data showed that fruit shape and number of seeds of *slapx4* mutant plants were similar to those of wild type plants (Figure 5E), suggesting that targeted mutagenesis of specific ascorbate degradation pathway would be less destructive than ectopic activation of the biosynthetic pathway in plants. In conclusion, the data presented here suggest that *SlAPX4* is a valuable candidate gene to engineer tomato plants for increasing ascorbate content in mature fruits without growth defects.

## Data Availability Statement

The datasets presented in this study can be found in online repositories. The names of the repository/repositories and accession number(s) can be found in the article/Supplementary Material.

## Author Contributions

JHD and YHJ planned and designed all the aspects of the study. JHD and SYP generated gene editing vectors and tomato transgenic plants. JHD, SYP, SHP, HMK, TDM, and SHM participated in the phenotypical analysis of the plant. JHD, JSS, and YHJ performed the data interpretation and wrote the manuscript. All authors contributed to the article and approved the submitted version of the manuscript.

## Funding

This study was supported by a grant from the New Breeding Technologies Development Program (Project No. PJ01487401 and PJ01654401) through the Rural Development Administration (RDA) and the National Research Foundation of Korea (NRF) grant funded by the Korean Government (MSIT) (NRF-2021R1F1A1060339 to JSS).

## Conflict of Interest

The authors declare that the research was conducted in the absence of any commercial or financial relationships that could be construed as a potential conflict of interest.

## Publisher's Note

All claims expressed in this article are solely those of the authors and do not necessarily represent those of their affiliated organizations, or those of the publisher, the editors and the reviewers. Any product that may be evaluated in this article, or claim that may be made by its manufacturer, is not guaranteed or endorsed by the publisher.

## References

[B1] Abdelgawad F. K. El-Mogy M. M. Mohamed I. a. M. Garchery C. Stevens G. R. (2019). Increasing ascorbic acid content and salinity tolerance of cherry tomato plants by suppressed expression of the ascorbate oxidase gene. Agronomy 9, 51. 10.3390/agronomy9020051

[B2] Abushita A. A. Hebshi E. A. Daood H. G. Biacs P. A. (1997). Determination of antioxidant vitamins in tomatoes. Food Chem. 60, 207–212. 10.1016/S0308-8146(96)00321-4

[B3] Agius F. González-Lamothe R. Caballero J. L. Muñoz-Blanco J. Botella M. A. Valpuesta V. (2003). Engineering increased vitamin C levels in plants by overexpression of a D-galacturonic acid reductase. Nat. Biotechnol. 21, 177–181. 10.1038/nbt777 12524550

[B4] Akram N. A. Shafiq F. Ashraf M. (2017). Ascorbic acid-A potential oxidant scavenger and its role in plant development and abiotic stress tolerance. Front. Plant Sci. 8, 613. 10.3389/fpls.2017.00613 28491070 PMC5405147

[B5] Badejo A. A. Wada K. Gao Y. Maruta T. Sawa Y. Shigeoka S.. (2012). Translocation and the alternative D-galacturonate pathway contribute to increasing the ascorbate level in ripening tomato fruits together with the D-mannose/L-galactose pathway. J. Exp. Bot. 63, 229–239. 10.1093/jxb/err275 21984649 PMC3245467

[B6] Beecher G. R. (1998). Nutrient content of tomatoes and tomato products. Proc. Soc. Exp. Biol. Med. 218, 98–100. 10.3181/00379727-218-44282a 9605204

[B7] Bulley S. Wright M. Rommens C. Yan H. Rassam M. Lin-Wang K.. (2012). Enhancing ascorbate in fruits and tubers through over-expression of the L-galactose pathway gene GDP-L-galactose phosphorylase. Plant Biotechnol. J. 10, 390–397. 10.1111/j.1467-7652.2011.00668.x 22129455

[B8] Caverzan A. Passaia G. Rosa S. B. Ribeiro C. W. Lazzarotto F. Margis-Pinheiro M. (2012). Plant responses to stresses: role of ascorbate peroxidase in the antioxidant protection. Genet. Mol. 35, 1011–1019. 10.1590/s1415-47572012000600016 23412747 PMC3571416

[B9] Chatzopoulou F. Sanmartin M. Mellidou I. Pateraki I. Koukounaras A. Tanou G.. (2020). Silencing of ascorbate oxidase results in reduced growth, altered ascorbic acid levels and ripening pattern in melon fruit. Plant Physiol. Biochem. 156, 291–303. 10.1016/j.plaphy.2020.08.040 32987259

[B10] Chen Z. Young T. E. Ling J. Chang S.-C. Gallie D. R. (2003). Increasing vitamin C content of plants through enhanced ascorbate recycling. Proc. Natl. Acad. Sci. U. S. A. 100, 3525–3530. 10.1073/pnas.0635176100 12624189 PMC152326

[B11] Conklin P. L. Norris S. R. Wheeler G. L. Williams E. H. Smirnoff N. Last R. L. (1999). Genetic evidence for the role of GDP-mannose in plant ascorbic acid (vitamin C) biosynthesis. Proc. Natl. Acad. Sci. U. S. A. 96, 4198–4203. 10.1073/pnas.96.7.4198 10097187 PMC22444

[B12] Di Matteo A. Sacco A. Anacleria M. Pezzotti M. Delledonne M. Ferrarini A.. (2010). The ascorbic acid content of tomato fruits is associated with the expression of genes involved in pectin degradation. BMC Plant Biol. 10, 163. 10.1186/1471-2229-10-163 20691085 PMC3095297

[B13] Dowdle J. Ishikawa T. Gatzek S. Rolinski S. Smirnoff N. (2007). Two genes in Arabidopsis thaliana encoding GDP-L-galactose phosphorylase are required for ascorbate biosynthesis and seedling viability. Plant J. 52, 673–689. 10.1111/j.1365-313X.2007.03266.x 17877701

[B14] Drouin G. Godin J.-R. Pagé B. (2011). The genetics of vitamin C loss in vertebrates. Curr. Genomics 12, 371–378. 10.2174/138920211796429736 22294879 PMC3145266

[B15] Dumville J. C. Fry S. C. (2003). Solubilisation of tomato fruit pectins by ascorbate: a possible non-enzymic mechanism of fruit softening. Planta 217, 951–961. 10.1007/s00425-003-1061-0 12838420

[B16] Engler C. Gruetzner R. Kandzia R. Marillonnet S. (2009). Golden gate shuffling: a one-pot DNA shuffling method based on type IIs restriction enzymes. PLoS ONE 4, e5553. 10.1371/journal.pone.0005553 19436741 PMC2677662

[B17] FAOSTAT. (2019). Statistics Devision of the Food and Agriculture Organization of the United Nations. Available online at: http://www.fao.org/faostat/en/#data (accessed November 8, 2021).

[B18] Fenech M. Amaya I. Valpuesta V. Botella M. A. (2019). Vitamin C content in fruits: Biosynthesis and regulation. Front. Plant Sci. 9, 2006. 10.3389/fpls.2018.02006 30733729 PMC6353827

[B19] Foyer C. H. Noctor G. (2011). Ascorbate and glutathione: the heart of the redox hub. Plant Physiol. 155, 2–18. 10.1104/pp.110.167569 21205630 PMC3075780

[B20] Gapper N. E. Mcquinn R. P. Giovannoni J. J. (2013). Molecular and genetic regulation of fruit ripening. Plant Mol. Biol. 82, 575–591. 10.1007/s11103-013-0050-3 23585213

[B21] Gatzek S. Wheeler G. L. Smirnoff N. (2002). Antisense suppression of l-galactose dehydrogenase in Arabidopsis thaliana provides evidence for its role in ascorbate synthesis and reveals light modulated l-galactose synthesis. Plant J. 30, 541–553. 10.1046/j.1365-313x.2002.01315.x 12047629

[B22] Gest N. Gautier H. Stevens R. (2013). Ascorbate as seen through plant evolution: the rise of a successful molecule? J. Exp. Bot. 64, 33–53. 10.1093/jxb/ers297 23109712

[B23] Gilbert L. Alhagdow M. Nunes-Nesi A. Quemener B. Guillon F. Bouchet B.. (2009). GDP-D-mannose 3,5-epimerase (GME) plays a key role at the intersection of ascorbate and non-cellulosic cell-wall biosynthesis in tomato. Plant J. 60, 499–508. 10.1111/j.1365-313X.2009.03972.x 19619161

[B24] Guo K. Li Z. Tian H. Du X. Liu Z. Huang H.. (2020). Cytosolic ascorbate peroxidases plays a critical role in photosynthesis by modulating reactive oxygen species level in stomatal guard cell. Front. Plant Sci. 11, 446–446. 10.3389/fpls.2020.00446 32457767 PMC7221183

[B25] Imai T. Ban Y. Terakami S. Yamamoto T. Moriguchi T. (2009). l-Ascorbate biosynthesis in peach: cloning of six l-galactose pathway-related genes and their expression during peach fruit development. Physiol. Plant. 136, 139–149. 10.1111/j.1399-3054.2009.01213.x 19453508

[B26] Ishikawa T. Maruta T. Yoshimura K. Smirnoff N. (2018). Biosynthesis and regulation of ascorbic acid in plants, in Antioxidants and Antioxidant Enzymes in Higher Plants, eds D. K. Gupta J. M. Palma F. J. Corpas. (Cham: Springer International Publishing), 163–179.

[B27] Jimenez A. Creissen G. Kular B. Firmin J. Robinson S. Verhoeyen M.. (2002). Changes in oxidative processes and components of the antioxidant system during tomato fruit ripening. Planta 214, 751–758. 10.1007/s004250100667 11882944

[B28] Koussevitzky S. Suzuki N. Huntington S. Armijo L. Sha W. Cortes D.. (2008). Ascorbate peroxidase 1 plays a key role in the response of Arabidopsis thaliana to stress combination. J. Biol. Chem. 283, 34197–34203. 10.1074/jbc.M806337200 18852264 PMC2590703

[B29] Laing W. A. Bulley S. Wright M. Cooney J. Jensen D. Barraclough D.. (2004). A highly specific L-galactose-1-phosphate phosphatase on the path to ascorbate biosynthesis. Proc. Natl. Acad. Sci. U. S. A. 101, 16976–16981. 10.1073/pnas.0407453101 15550539 PMC534719

[B30] Li C. Liu Y. Liu X. Mai K. K. K. Li J. Guo X.. (2021). Chloroplast thylakoid ascorbate peroxidase PtotAPX plays a key role in chloroplast development by decreasing hydrogen peroxide in *Populus tomentosa*. J. Exp. Bot. 72, 4333–4354. 10.1093/jxb/erab173 33884422

[B31] Li M. Ma F. Liang D. Li J. Wang Y. (2010). Ascorbate biosynthesis during early fruit development is the main reason for its accumulation in kiwi. PLoS ONE 5, e14281. 10.1371/journal.pone.0014281 21151561 PMC3000333

[B32] Li X. Ye J. Munir S. Yang T. Chen W. Liu G.. (2019). Biosynthetic gene pyramiding leads to ascorbate accumulation with enhanced oxidative stress yolerance in tomato. Int. J. Mol. Sci. 20, 1558. 10.3390/ijms20071558 30925709 PMC6480547

[B33] Lima-Silva V. Rosado A. Amorim-Silva V. Muñoz-Mérida A. Pons C. Bombarely A.. (2012). Genetic and genome-wide transcriptomic analyses identify co-regulation of oxidative response and hormone transcript abundance with vitamin C content in tomato fruit. BMC Genomics 13, 187. 10.1186/1471-2164-13-187 22583865 PMC3462723

[B34] Liu J.-X. Feng K. Duan A.-Q. Li H. Yang Q.-Q. Xu Z.-S.. (2019). Isolation, purification and characterization of an ascorbate peroxidase from celery and overexpression of the AgAPX1 gene enhanced ascorbate content and drought tolerance in Arabidopsis. BMC Plant Biol. 19, 488. 10.1186/s12870-019-2095-1 31711410 PMC6849298

[B35] Locato V. Cimini S. Gara L. D. (2013). Strategies to increase vitamin C in plants: from plant defense perspective to food biofortification. Front. Plant Sci. 4, 152. 10.3389/fpls.2013.00152 23734160 PMC3660703

[B36] Macknight R. C. Laing W. A. Bulley S. M. Broad R. C. Johnson A. A. Hellens R. P. (2017). Increasing ascorbate levels in crops to enhance human nutrition and plant abiotic stress tolerance. Curr. Opin. Biotechnol. 44, 153–160. 10.1016/j.copbio.2017.01.011 28231513

[B37] Marquez L. A. Quitoriano M. Zilinskas B. A. Dunford H. B. (1996). Kinetic and spectral properties of pea cytosolic ascorbate peroxidase. FEBS Lett. 389, 153–156. 10.1016/0014-5793(96)00562-5 8766820

[B38] Maruta T. Yonemitsu M. Yabuta Y. Tamoi M. Ishikawa T. Shigeoka S. (2008). Arabidopsis phosphomannose isomerase 1, but not phosphomannose isomerase 2, is essential for ascorbic acid biosynthesis. J. Biol. Chem. 283, 28842–28851. 10.1074/jbc.M805538200 18755683 PMC2661998

[B39] Mellidou I. Keulemans J. Kanellis A. K. Davey M. W. (2012). Regulation of fruit ascorbic acid concentrations during ripening in high and low vitamin C tomato cultivars. BMC Plant Biol. 12, 239. 10.1186/1471-2229-12-239 23245200 PMC3548725

[B40] Mellidou I. Koukounaras A. Kostas S. Patelou E. Kanellis A. K. (2021). Regulation of vitamin C accumulation for improved tomato fruit quality and alleviation of abiotic stress. Genes 12, 694. 10.3390/genes12050694 34066421 PMC8148108

[B41] Najami N. Janda T. Barriah W. Kayam G. Tal M. Guy M.. (2008). Ascorbate peroxidase gene family in tomato: its identification and characterization. Mol. Genet. Genomics 279, 171–182. 10.1007/s00438-007-0305-2 18026995

[B42] Paciolla C. Fortunato S. Dipierro N. Paradiso A. De Leonardis S. Mastropasqua L.. (2019). Vitamin C in plants: from functions to biofortification. Antioxidants 8, 519. 10.3390/antiox8110519 31671820 PMC6912510

[B43] Parsons H. T. Yasmin T. Fry S. C. (2011). Alternative pathways of dehydroascorbic acid degradation *in vitro* and in plant cell cultures: novel insights into vitamin C catabolism. Biochem. J. 440, 375–383. 10.1042/bj20110939 21846329

[B44] Pnueli L. Liang H. Rozenberg M. Mittler R. (2003). Growth suppression, altered stomatal responses, and augmented induction of heat shock proteins in cytosolic ascorbate peroxidase (*Apx1*)-deficient *Arabidopsis* plants. Plant J. 34, 187–203. 10.1046/j.1365-313X.2003.01715.x 12694594

[B45] Qian W. Yu C. Qin H. Liu X. Zhang A. Johansen I. E.. (2007). Molecular and functional analysis of phosphomannomutase (PMM) from higher plants and genetic evidence for the involvement of PMM in ascorbic acid biosynthesis in Arabidopsis and *Nicotiana benthamiana*. Plant J. 49, 399–413. 10.1111/j.1365-313X.2006.02967.x 17217471

[B46] Sacco A. Raiola A. Calafiore R. Barone A. Rigano M. M. (2019). New insights in the control of antioxidants accumulation in tomato by transcriptomic analyses of genotypes exhibiting contrasting levels of fruit metabolites. BMC Genomics 20, 43. 10.1186/s12864-019-5428-4 30646856 PMC6332538

[B47] Sato S. Tabata S. Hirakawa H. E. A. (2012). The tomato genome sequence provides insights into fleshy fruit evolution. Nature 485, 635–641. 10.1038/nature11119 22660326 PMC3378239

[B48] Shigeoka S. Ishikawa T. Tamoi M. Miyagawa Y. Takeda T. Yabuta Y.. (2002). Regulation and function of ascorbate peroxidase isoenzymes. J. Exp. Bot. 53, 1305–1319. 10.1093/jexbot/53.372.1305 11997377

[B49] Tyagi S. Shumayla Verma P. C. Singh K. Upadhyay S. K. (2020). Molecular characterization of ascorbate peroxidase (APX) and APX-related (APX-R) genes in *Triticum aestivum L*. Genomics 112, 4208–4223. 10.1016/j.ygeno.2020.07.023 32681868

[B50] Wheeler G. Ishikawa T. Pornsaksit V. Smirnoff N. (2015). Evolution of alternative biosynthetic pathways for vitamin C following plastid acquisition in photosynthetic eukaryotes. Elife 4, e06369. 10.7554/eLife.06369 25768426 PMC4396506

[B51] Wheeler G. L. Jones M. A. Smirnoff N. (1998). The biosynthetic pathway of vitamin C in higher plants. Nature 393, 365–369. 10.1038/30728 9620799

[B52] Wu B. Wang B. (2019). Comparative analysis of ascorbate peroxidases (APXs) from selected plants with a special focus on *Oryza sativa* employing public databases. PLoS ONE 14, e0226543. 10.1371/journal.pone.0226543 31856232 PMC6922425

[B53] Yin B. Zhang J. Liu Y. Pan X. Zhao Z. Li H.. (2019). *PtomtAPX*, a mitochondrial ascorbate peroxidase, plays an important role in maintaining the redox balance of *Populus tomentosa Carr*. Sci. Rep. 9, 19541. 10.1038/s41598-019-56148-w 31862975 PMC6925217

[B54] Zhang G.-Y. Liu R.-R. Zhang C.-Q. Tang K.-X. Sun M.-F. Yan G.-H.. (2015). Manipulation of the rice L-Galactose pathway: evaluation of the effects of transgene overexpression on ascorbate accumulation and abiotic stress tolerance. PLoS ONE 10, e0125870. 10.1371/journal.pone.0125870 25938231 PMC4418601

[B55] Zhang Y.-Y. Li H.-X. Shu W.-B. Zhang C.-J. Ye Z.-B. (2011). RNA interference of a mitochondrial APX gene improves vitamin C accumulation in tomato fruit. Sci. Hortic. 129, 220–226. 10.1016/j.scienta.2011.03.025

