# Supplementary material for: Development of a Genome-Edited Tomato With High Ascorbate Content During Later Stage of Fruit Ripening Through Mutation of SlAPX4
Source: Front Plant Sci. 2022 Apr 12;13:836916. doi: 10.3389/fpls.2022.836916 (PMC9039661; doi:10.3389/fpls.2022.836916)
Supplement: Supplementary file 1 [file Table_1.DOCX]

**Supplementary Table 1.** Primers used in this study**.**

| **Primer name** | **Sequence(5'→3')** | **Purpose** |
| --- | --- | --- |
| NPTII-F | AACAAGATGGATTGCACGCA | Transgenic plant Screening |
| NPTII-R | AAGAAGGCGATAGAAGGCGA |  |
| Cas9-300end-F | GACGCTAACCTCGATAAGGT |  |
| NOS-R | CAAGACCGGCAACAGGATTCAATC |  |
| APX Set 2-F | CTTGGTACTAGTCGTAATGTG | Amplification of the target region (*SlAPX4*) |
| APX Set 2-R | GAAACCATGTATTGCATCTTC |  |
| APX4-sgRNA1 | TGTGGTCTCAATTTTTGGAAATCGACGTTTGATGTTTTAGAGCTAGAAATAGCAAG | gRNA1 amplification |
| APX4-sgRNA2 | TGTGGTCTCAATTAAGCAGTTGAAAAATGTAAGGTTTTAGAGCTAGAAATAGCAAG | gRNA2 amplification |
| APX4-sgRNA3 | TGTGGTCTCAATTGTTAAGAATTTTTTACATGAGTTTTAGAGCTAGAAATAGCAAG | gRNA3 amplification |
| APX4-sgRNA4 | TGTGGTCTCAATTGATGTCAAAACCAAAACTGGGTTTTAGAGCTAGAAATAGCAAG | gRNA4 amplification |
| CRISPR UNI-R | TGTGGTCTCAAGCGTAATGCCAACTTTGTAC | gRNA amplification: universal |
| SlAPX4-RT-F | GACGTTTGATCGGAGAAGAAATGGTG | *SlAPX4* gene amplification |
| SlAPX4-RT-R | CGGGTGCCTGATTGTTCCAA |  |
| SlAPX1-RT-F | TGCTGAAGCTTAAGATGCTGATTG | *SlAPX1* gene amplification |
| SlAPX1-RT-R | CATCGTCTAACGTAGCTGCC |  |
| SlAPX2-RT-F | TCAGATCAACTACTATGGGCAAGTC | *SlAPX2* gene amplification |
| SlAPX2-RT-R | CCACCAGTTTTGGAACACACATCATA |  |
| SlAPX3-RT-F | CGATCGGTTCTCCAATGGCGA | *SlAPX3* gene amplification |
| SlAPX3-RT-R | AGAACCATTGGGTCCACCAGTC |  |
| Tomato actin-R2 | CTCTTCTGTCTGGCTACACCATC | *Tomato actin* gene amplification |
| Tomato actin-L2 | AGTATGATGAGTCTGGTCCTTCCA |  |
| SlGMP-RT-F | ACTCACCCTCAGCGTCCCA | Expression of ascorbate biosynthetic gene  (*SlGMP*, Solyc03g096730) |
| SlGMP-RT-R | CTTGATTCCAAGGCTTGCCTC |  |
| SlGLDH-RT-F | GTGGGACTCATGAGGTTCGG | Expression of ascorbate biosynthetic gene  (*SlGLDH*, Solyc10g079470) |
| SlGLDH-RT-R | CGCCAAATTCACCATCCCAG |  |
| SlGME1-RT-F | ATTGGAAGCCAATCCATCTG | Expression of ascorbate biosynthetic gene  (*SlGME1*, Solyc01g097340) |
| SlGME1-RT-R | AAACCAAGAAACGCCAACAA |  |
| SlGME2-RT-F | TTGTTCGCGTGGAATGTTTA | Expression of ascorbate  biosynthetic gene  (*SlGME2*, Solyc09g082990) |
| SlGME2-RT-R | GTAGGTGCAATGAGGGGATG |  |
| SlDHAR1-RT-F | AACTGTACCATCTCGAGGTGG | Expression of ascorbate  biosynthetic gene  (*SlDHAR1*, Solyc05g054760) |
| SlDHAR1-RT-R | TCGAGTCAGTCATACGTTAAACCT |  |
| SlDHAR2-RT-F | CACGAAGTTCAGAGCACCCA | Expression of ascorbate  biosynthetic gene  (*SlDHAR2*, Solyc11g011250) |
| SlDHAR2-RT-R | CCAGTCAGGCTTGTTACTCAAG |  |
| SlMDHAR-RT-F | TGCACCATATGAACGTCCAGC | Expression of ascorbate  biosynthetic gene  (*SlMDHAR*, Solyc08g081530) |
| SlMDHAR-RT-R | GCTTTCCTGAATTTGTTGTCAGAG |  |
