# Supplementary figures and images for: Development of a Genome-Edited Tomato With High Ascorbate Content During Later Stage of Fruit Ripening Through Mutation of SlAPX4
Source: Front Plant Sci. 2022 Apr 12;13:836916. doi: 10.3389/fpls.2022.836916 (PMC9039661; doi:10.3389/fpls.2022.836916)

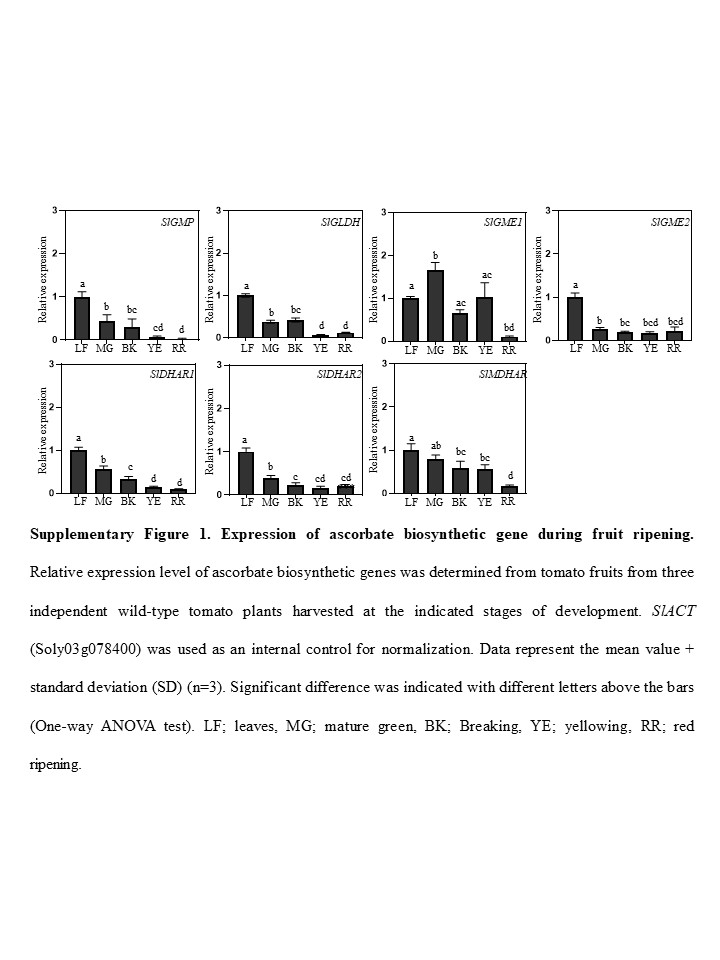

Supplement: Supplementary file 2 [file Image_1.JPEG]

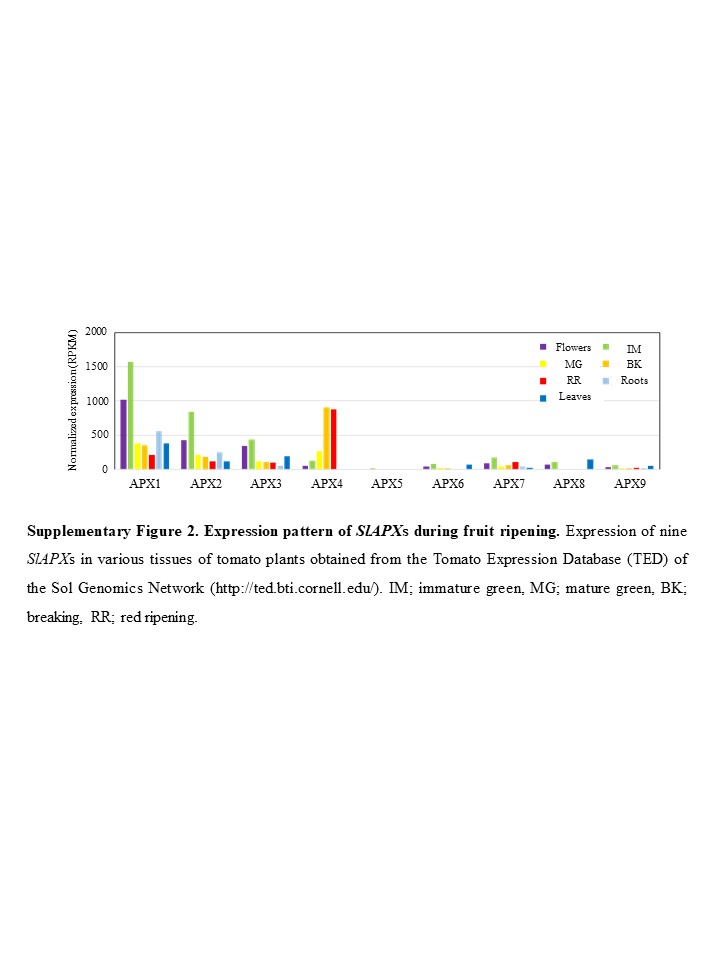

Supplement: Supplementary file 3 [file Image_2.JPEG]

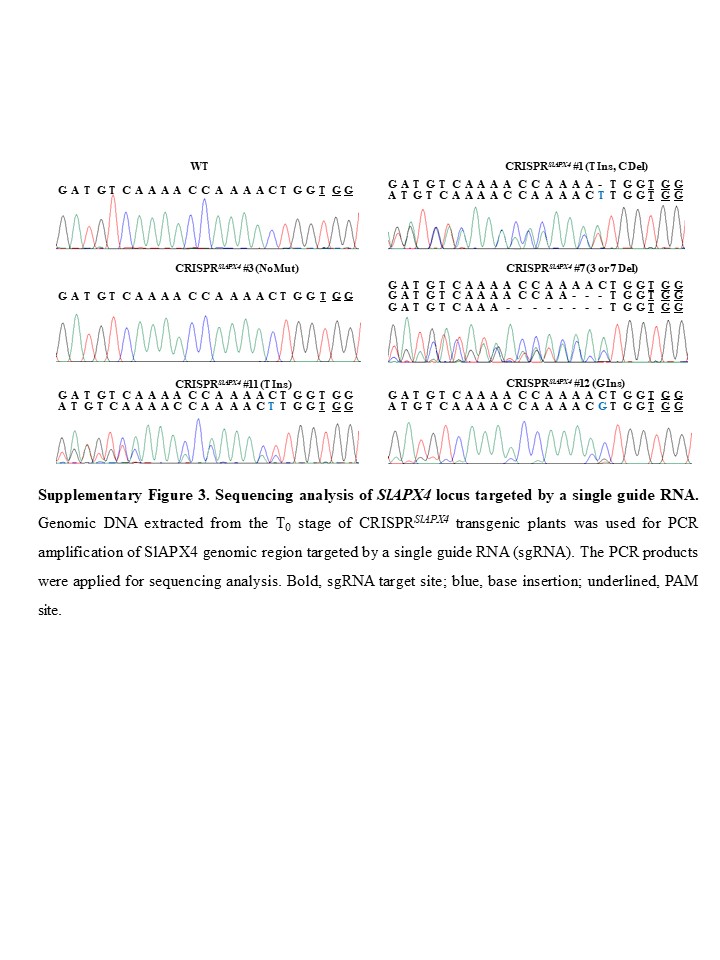

Supplement: Supplementary file 4 [file Image_3.JPEG]

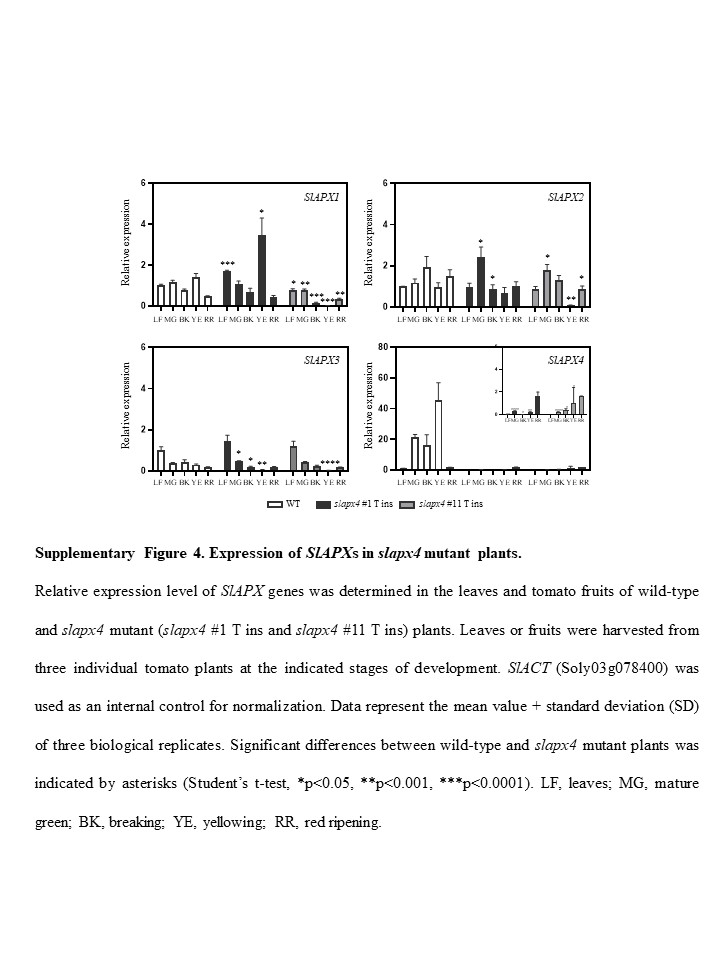

Supplement: Supplementary file 5 [file Image_4.JPEG]

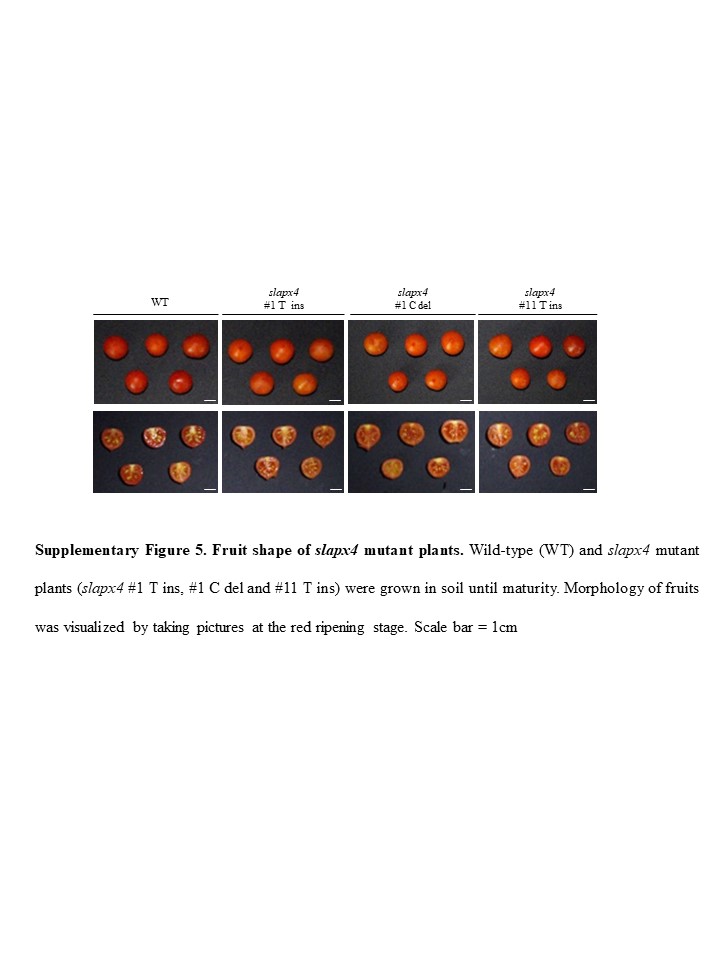

Supplement: Supplementary file 6 [file Image_5.JPEG]
